# Early expansion of activated adaptive but also exhausted NK cells during acute severe SARS-CoV-2 infection

**DOI:** 10.3389/fcimb.2023.1266790

**Published:** 2023-08-30

**Authors:** Maren Claus, Naomi Pieris, Doris Urlaub, Peter Bröde, Bernhard Schaaf, Deniz Durak, Frank Renken, Carsten Watzl

**Affiliations:** ^1^ Department for Immunology, Leibniz Research Centre for Working Environment and Human Factors (IfADo) at TU Dortmund, Dortmund, Germany; ^2^ Department of Respiratory Medicine and Infectious Diseases, Klinikum Dortmund, Dortmund, Germany; ^3^ Faculty of Health, University Witten/Herdecke, Herdecke, Germany; ^4^ Dortmund Health Department, Dortmund, Germany

**Keywords:** natural killer (NK) cell, SARS-CoV-2 infection, immunophenotyping, exhaustion, COVID-19

## Abstract

The analysis of immunological parameters during the course of a SARS-CoV-2 infection is of great importance, both to identify diagnostic markers for the risk of a severe course of COVID-19, and to better understand the role of the immune system during the infection. By using multicolor flow cytometry we compared the phenotype of Natural Killer (NK) cells from hospitalized COVID-19 patients during early SARS-CoV-2 infection with samples from recovered and SARS-CoV-2 naïve subjects. Unsupervised high-dimensional analysis of 28-color flow cytometric data revealed a strong enrichment of NKG2C expressing NK cells in response to the acute viral infection. In addition, we found an overrepresentation of highly activated NK cell subsets with an exhausted phenotype. Moreover, our data show long-lasting phenotypic changes within the NK cell compartment that did not completely reverse up to 2 months after recovery. This demonstrates that NK cells are involved in the early innate immune response against SARS-CoV-2.

## Introduction

The immune system is involved in the control of SARS-CoV-2 infections, but also in the pathogenesis of COVID-19. While early and effective immune responses are important to control the infection and to prevent severe disease (Bastard et al., 2020; Hadjadj et al., 2020; Zhang et al., 2020; Kramer et al., 2021; Witkowski et al., 2021), an exaggerated immune response can also contribute to increased inflammation and severe COVID-19 (Hadjadj et al., 2020; Malengier-Devlies et al., 2022). During the early phase of the COVID-19 pandemic there was only little information about the immune response to SARS-COV-2 infection. Several changes in leukocyte function and phenotype were described in COVID-19 patients. An early analysis reports an increase in neutrophilic granulocytes (Chen et al., 2020). In contrast, lymphocyte counts were decreased in 35% of patients. Inflammatory markers IL-6 and CRP were elevated in 52% and 86% of patients, respectively. Further studies correlated these findings with the severity of infection (Qin et al., 2020; Wan et al., 2020; Wang et al., 2020). Especially in severe COVID-19, the numbers of lymphocytes, especially T and NK cells were decreased. Similarly, the increase of inflammatory markers such as CRP, IL-6, and TNF was more pronounced in the patients with severe courses of the infection. This demonstrated that an exaggerated immune response with the accompanying strong inflammatory reaction contributes to the pathology of COVID-19. Similarly, the immune response to the virus appears to decrease the number of cytotoxic T cells and NK cells, and a strong decrease was associated with poorer viral control (Qin et al., 2020; Wang et al., 2020). However, these parameters appear to return to normal as patients recover (Wan et al., 2020; Wang et al., 2020). There were also immune responses that were associated with successful viral suppression and mild disease. For example, antibody-producing cells and specific T helper cells have been described in the blood of a patient with a mild course of COVID-19 (Thevarajan et al., 2020). At the same time, activation was evident in cytotoxic T cells. These positive signs of an immune response were then correlated with the patient’s recovery and the appearance of virus-specific antibodies.

Early studies also looked more closely at the phenotype of T and NK cells during SARS-CoV-2 infection. Despite non-increased inflammation levels, normal neutrophil and lymphocyte numbers, increased markers of exhaustion were detected in T cells (Zheng et al., 2020a). Thus, the expression of the inhibitory receptors PD-1, CTLA-4, and TIGIT was increased. Another early study showed increased expression of the inhibitory receptor NKG2A on NK cells which was paralleled by decreased levels of T and NK cells (Zheng et al., 2020b). These results indicated a reduction of T and NK cell effector functions during the immune response against SARS-CoV-2. This could be causally related to the decrease in T and NK cell numbers in the blood of patients with severe course, but also to exhaustion of remaining T and NK cells. Interestingly, not only did the numbers of T and NK cells recover in recovered patients, but the expression of NKG2A also decreased again on these cells (Zheng et al., 2020b).

These early findings of immune system alterations during mild and severe COVID-19 demonstrated the importance of analyzing immunological parameters during the course of a SARS-CoV-2 infection, both to identify diagnostic markers and to better understand the role of the immune system in the infection. For this purpose, we compared NK cells from acutely infected, hospitalized COVID-19 patients with samples from recovered and SARS-CoV-2 naïve subjects. Here we describe phenotypical alterations of NK cells from COVID-19 patients that were hospitalized during the first year of the COVID-19 pandemic. Multicolor flow cytometry revealed the strong enrichment of NKG2C expressing cells in response to SARS-CoV-2 infection. In addition, we found an overrepresentation of highly activated NK cell subsets which show an exhausted phenotype. Moreover, there is evidence of long-lasting phenotypic changes that did not completely reverse up to 2 months after recovery.

## Materials and methods

### Participants and study design

The study was approved by the local ethics committees (#178, IfADo and #301-2008, CAPNETZ, Hannover Medical School). All participants gave written informed consent.

A total of 50 COVID-19 patients admitted to Klinikum Nord Dortmund between April 2020 and January 2021 were enrolled in the study and blood was analyzed for leukocyte count, serum CRP and anti-Spike RBD IgM, IgA and IgG at the day of admission to the hospital, during the course of the disease (every 3 days), and on the day of discharge. A total of 32 patients with samples from at least 3 visits (COV A-C) were included in the study. We further analyzed samples from 32 healthy controls (HC), which were collected in 2019 before the onset of SARS-CoV-2 pandemic, and 19 samples from subjects who had recovered (REC) from previous SARS-CoV-2 infection. REC samples were taken in March and April 2020 (55 ± 6.6 days after infection; range 48 - 71 days). Demographic information of all participants is shown in **Table 1**. Of the REC sample, only one subject was hospitalized for 3 days, but not admitted to ICU. SARS-CoV-2 infection was determined by PCR. Date of infection was not reported, therefore we used the date of positive PCR test as day 0 (Urlaub et al., 2022). Clinical parameters of acutely infected COVID-19 patients are summarized in **Table 2**.

**Table 1:** Distribution of age and sex by group.

	HC, N = 32^1^	REC, N = 19^1^	COV, N = 32^1^	p-value^2^
Age	46 ± 14 (24–70)	46 ± 9 (27–62)	56 ± 14 (34–84)	0.0056
missing	0	2	0	
Sex				0.024
f	9 (28%)	10 (63%)	8 (25%)	
m	23 (72%)	6 (37%)	24 (75%)	
missing	0	3	0	

^1^Mean ± SD (range).

^2^Kruskal-Wallis rank sum test or Chi-square test

Age and sex distribution of the study participants in the samples of recovered patients (REC) and healthy controls (HC) compared to hospitalized COVID-19 patients (COV). Statistical significance of the overall difference between group means was assessed for age by the Kruskal-Wallis rank sum test, and for sex by the Chi-squared test.

**Table 2:** Clinical parameters by visit.

Parameter	A, N = 32^1^	B, N = 32^1^	C, N = 32^1^	p-value^2^
CRP (mg/L)	68 ± 52 (3)	40 ± 45 (3)	28 ± 44 (3)	<0.0001
Leukocytes per nL blood	8.0 ± 4.7 (1)	9.4 ± 6.1 (1)	10.9 ± 4.7 (1)	<0.0003
IgM (rel. absorbance)	4.5 ± 4.2 (1)	6.6 ± 3.8 (1)	7.8 ± 3.7 (1)	<0.0001
IgA (rel. absorbance)	2.2 ± 2.1 (1)	3.6 ± 2.3 (1)	4.1 ± 2.1 (1)	<0.0001
IgG (rel. absorbance)	2.9 ± 3.4 (1)	5.2 ± 3.3 (1)	7.2 ± 2.8 (1)	<0.0001
days since symptom onset	8.1 ± 4.5 (4) (range 1–22)			

^1^Mean ± SD (N missing).

^2^Friedman rank sum test.

Means and standard deviations (SD) and number of missing values of days from symptom onset to hospitalization (visit A), the concentration of C-reactive protein (CRP), number of leukocytes per nL blood and serum concentration of anti-Spike RBD IgM, IgA and IgG measured at visits A to C for hospitalized COVID-19 patients. Statistical significance of the overall difference between group means was assessed by the Friedman rank sum test.

Samples were taken from venous blood and collected in appropriate tubes (Serum and EDTA Monovettes, Sarstedt) and plasma and serum was stored at -80°C until use.

PBMC were isolated by Ficoll density centrifugation and cryopreserved at -170°C until use.

### Flow cytometry

All antibodies were individually titrated to determine the optimal dilution. All antibodies and dilutions are listed in **suppl. Table 1**. PBMC were used immediately after thawing and were kept on ice during the staining procedure, unless stated otherwise. For each sample, 0.5 × 10^6^ cells were stained with the live/dead stain Zombie NIR in PBS for 20 min at RT and washed with PBS/2% human serum. Afterwards, samples were stained with 50 µl of the antibody cocktail for 20 min at 4°C in the dark and then washed with FACS buffer (PBS/2% FCS). Cells were resuspended in 150 μl FACS buffer and kept on ice until analysis at the same day on a 5 laser Cytek^®^ Aurora (Cytek^®^ Biosciences). Data were analyzed using the FlowJo software (version 10.8.2; FlowJo LLC, USA) incl. the plugins FlowAI, DownSample, tSNE (optSNE), Phenograph and Cluster Explorer for high parameter analysis.

Principal component analysis (PCA) and statistical group comparisons were performed with GraphPad Prism 9.

### Quantification of anti-Spike RBD antibodies

Antibodies against Spike-RBD were quantified as described (Urlaub et al., 2021; Urlaub et al., 2022). Briefly, the RBD sequence (spike glycoprotein amino acids 319–541) of the Wuhan strain of SARS-CoV-2 with a C-terminal HIS-tag was expressed in HEK 293-F cells and purified on a HisTrap Excel column using an ÄKTAxpress purification system. Ninety-six well flat bottom plates (maxisorp; Nunc) were coated with 3 μg/mL of SARS-CoV-2 spike RBD overnight at 4°C. Plates were washed, blocked with Biolegend ELISA diluent, and then incubated with serum samples diluted in blocking buffer. The S Antibody (humanized anti-Spike antibody by Dianova/Cusabio, Stock concentration: 0.3 mg/mL) was used as positive control (1:5 000 final dilution) and calibrator (1:40 000 final dilution) for the measurement of IgG. The same patient serum was included at a dilution of 1:800 in each assay as calibrator for quantification of IgM and IgA. 172 healthy control sera collected before the emergence of SARS-CoV-2 were tested to confirm the specificity of the assay (>95% for IgM [6/172] and 100% for IgA and IgG [0/172]). As a secondary antibody, HRP conjugated anti-human IgG, IgA or IgM (Dianova) was used and signals were detected with 1 Step Ultra TMB (Pierce). The relative absorbance was calculated using the formula: (sample - negative control)/(calibrator - negative control). Values >1 are considered positive.

## Results

To analyze immunological parameters during the early course of a severe SARS-CoV-2 infection we collected blood from COVID-19 patients (COV) that were hospitalized between April 2020 and January 2021, before the emergence of SARS-CoV-2 variants of concern. We collected samples during admission (sample A), at day four (sample B) and at day seven (sample C) of the hospital stay. Additionally, we collected blood samples from individuals about 2 months after their recovery from a SARS-CoV-2 infection (REC) and used samples from SARS-Cov-2 naïve individuals as healthy controls (HC) (see material and methods for details). To gain detailed information about the effects of SARS-CoV-2 infection on different lymphocyte subsets we isolated PBMCs from the blood samples and used high dimensional 28-color flow cytometry to investigate markers of lymphocyte activation, exhaustion and senescence. All antigens and respective antibodies are summarized in **suppl. Table 1**.

**Figure 1:**
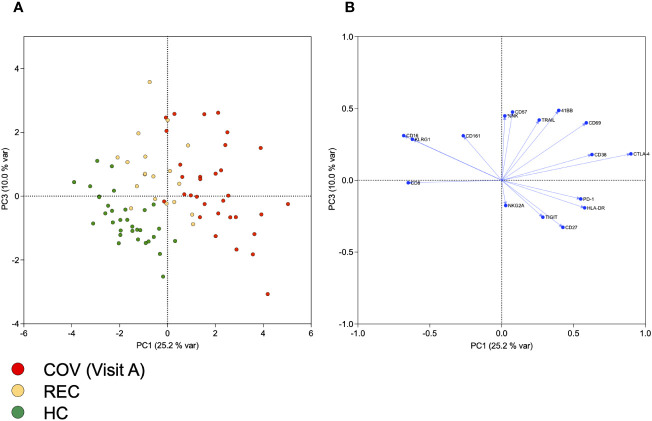
Principal component analysis of NK cell frequency and of geometric means of markers expressed on NK cells. **(A)** Scores of individual samples from COV (visit A), REC and HC for PCs 1 and 3, together explaining 35% of variance. **(B)** Loadings of variables included in PCA on PCs 1 and 3.

First, we determined the intensity (geometric Mean (geoMean)) and the frequency of expression for individual markers on B, T and NK cells. Principal component analysis (PCA) of the results allows a clear discrimination of samples from acutely infected patients and healthy controls. Interestingly, samples from recovered subjects cluster between the other two groups, indicating long-lasting immunological alterations and a gradual recovery of the lymphocyte phenotype after resolution of the acute infection (**suppl. Figure 1**). Previous work suggested a strong influence of SARS-CoV-2 infection on NK cell numbers and function. Therefore, we performed a detailed flow cytometric analysis of NK cell phenotype in terms of activation, exhaustion, and senescence (see **suppl. Figure 2** for gating strategy). We determined surface expression levels by geoMean and the frequency of NK cells positive for selected markers. A PCA of geoMeans and NK cell frequency revealed three clusters progressing from patients via recovered subjects to healthy controls (**Figure 1A**). This demonstrates that similar to the analysis of all lymphocyte subsets, there are specific changes within the NK cell compartment upon SARS-CoV-2 infection. PC1, which explains 25.2% of variance, is particularly useful for differentiating COV from HC. COV samples score high for PC1, which is characterized by high loadings of activation markers CD38, HLA-DR and CD69, but also TIGIT, CTLA-4, and PD-1 and negative loading of CD16 and CD161 (**Figure 1B** and **suppl. Table 2**). Interestingly, in combination with PC3, which is mainly characterized by high loadings of CD57 and KLRG1 and NK cell frequency, we were able to differentiate the REC samples from COV and HC.

**Figure 2:**
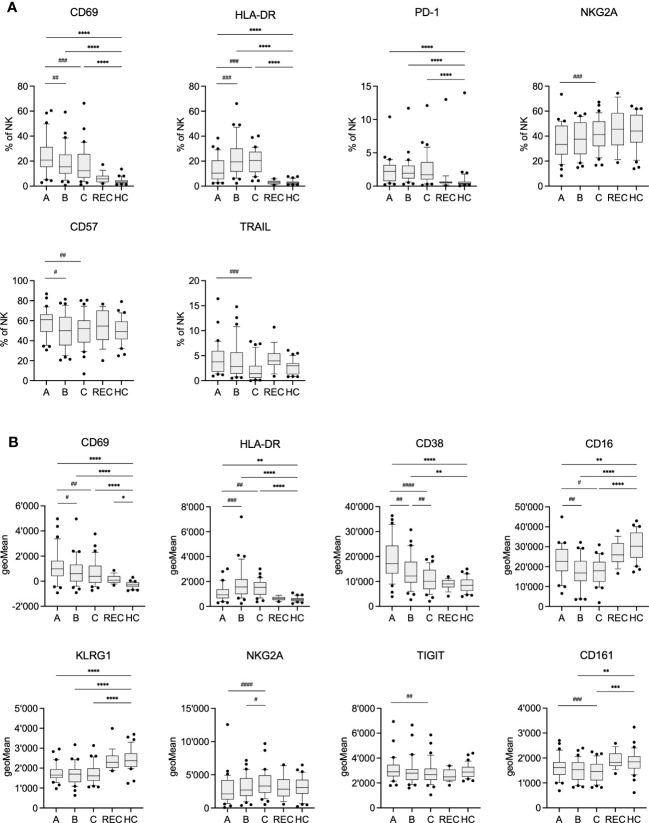
Frequency **(A)** and geometric Mean **(B)** of selected markers on NK cells of COV visits A-C, REC and HC. Pairwise comparisons with HC were performed using the Kruskal-Wallis test with Dunn’s post test. (*:p<.05, **:p<.01, ***:p<.001, ****:p<.0001). Pairwise comparisons between visits A-C were performed using the Friedman repeated measures test with Dunn’s post test. (#:p<.05, ##:p<.01, ###:p<.001, ####:p<.0001).

We also analyzed other clinical parameters in the COV group such as CRP, leukocyte count and the amount of IgM, IgA and IgG antibodies specific for SARS-CoV-2 Spike RBD (**Table 2** and **suppl. Figure 3**). Comparing samples A-C demonstrated that CRP and leukocyte counts significantly improved during the hospital stay. Similarly, Spike RBD specific IgM, IgA and IgG antibodies increased during the same time, indicative of the beginning adaptive immune response. However, we were not able to separate samples A-C using PCA of data derived from flow cytometry (data not shown). Therefore, we compared frequency and expression levels of individual markers on NK cells of COV samples A-C, REC and HC, to get an insight into the kinetics of the NK cell phenotype during disease progression (**Figure 2**). As expected, in COV samples we found high frequencies of NK cells expressing the activation markers CD69 and HLA-DR and the exhaustion marker PD-1 (**Figure 2A**), which was paralleled by increased expression levels (geoMeans) of the activation markers CD69, CD38 and HLA-DR. In contrast, the expression of CD16 and KLRG1 was decreased in COV samples compared to REC and HC (**Figure 2B**). This demonstrates that NK cells are highly activated during the early phase of COVID-19 as sample A was taken 8.1 ± 4.5 (range 1-22) days after sympton onset. Additionally, in the hospitalized patients we already find some markers for NK cell exhaustion. We did not find significant differences between the REC and HC groups with the exception of a small but significant difference in the mean expression levels of CD69, which is of unknown biological significance.

The activation markers CD69, TRAIL and CD38 significantly decreased during the hospital stay, which paralleled the decreasing CRP levels and elevated concentrations of anti-Spike RBD antibodies in serum (**Table 2** and **suppl. Figure 3**). However, the frequency of PD-1 and HLA-DR expressing cells remained stable at high levels. Similarly, expression of KLRG1, CD161 and CD16 remained low or even decreased further. In contrast to previous findings, we observed only small, yet significant, increase in NKG2A expression from sample A to C. However, expression of NKG2A in COV samples was not significantly different from HC. Although the results of PCA indicate an intermediate phenotype of the REC samples, individual markers were very similar to the values of HC. Therefore, there is not just a single marker which could explain the separation of the REC from the HC samples in the PCA.

**Figure 3:**
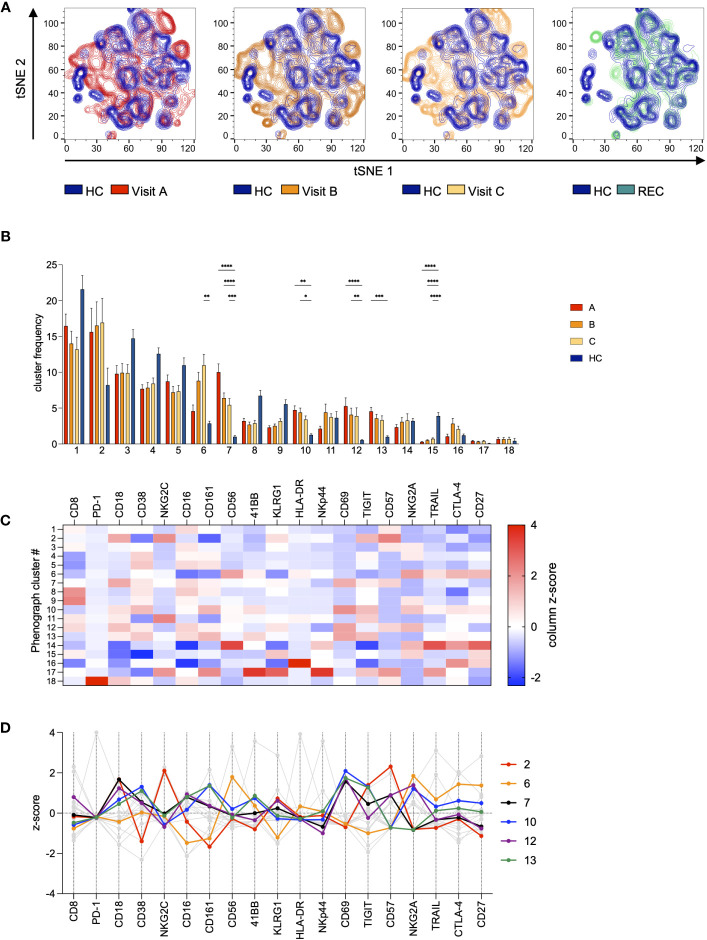
Unsupervised high-dimensional analysis of NK cells. All samples were gated on NK cells and then concatenated. Data was subjected to dimensionality reduction by tSNE **(A)** and cluster analysis using Phenograph **(B–D)**. **(A)** Overlay of tSNE plots of COV visits A-C (red/orange) and REC (green) with HC (blue). **(B)** Frequency distribution of Phenograph clusters in COV visits A-C and HC. Data are shown as mean ± SEM. Pairwise comparisons with HC were performed using the Kruskal-Wallis test with Dunn’s post test. (*:p<.05, **:p<.01, ***:p<.001, ****:p<.0001). **(C)** Features of all Phenograph clusters. Data are shown as z-scores of MFI. **(D)** Features of Phenograph clusters overrepresented in COV visits A-C. Data are shown as z-scores of MFI.

Next, we analyzed all data in parallel in an unsupervised high-dimensional analysis approach to investigate the distribution of specific NK cell subsets during the course of the infection. We used FlowJo and included plugins tSNE for dimensionality reduction and the clustering algorithm Phenograph (**suppl. Figure 4**), to identify NK cell subsets, that are specifically altered in COV samples or that change during disease progression. When we overlayed the samples from HC and the samples from A, B, and C from the COV patients we saw several differences in the subset distribution of the NK cells (**Figure 3A**). More specifically, there were several NK cell subsets that seemed to be specifically enriched in the COV samples. Phenograph analysis identified 18 clusters within the NK cells. As cluster frequency was not significantly different between REC and HC (data not shown), we decided to exclude REC samples from the further analyses. Five of these clusters were significantly overrepresented in COV samples compared to HC (**Figure 3B**). Cluster 2 was the most prominent cluster in the COV samples, and it remained stable over the course of disease (samples A-C, **suppl. Figure 5**). It is characterized by high levels of NKG2C and CD57 and low expression of CD38 (**Figures 3C, D**, and **suppl. Figure 6**). Therefore, this cluster is reminiscent of differentiated, adaptive NK cells. The frequency of cluster 6 significantly increased during the course of the disease (**suppl. Figure 5**). It is identified as CD56^bright^ NK cells with high expression of NKG2A, CD27, HLA-DR and TRAIL and low levels of CD16 and CD161, reminiscent of activated CD56^bright^ NK cells. The remaining four significantly overrepresented clusters 7, 10, 12, and 13 showed a tendency of decrease during the course of the disease and they are mainly defined by high expression of the activation marker CD69. In addition, these clusters are characterized by a varying expression of markers for exhaustion and senescence. In more detail, NK cells in clusters 7 and 12 are among the most mature NK cells, as indicated by high expression of CD57. The high expression level of NKG2A in addition to CD57 in cluster 12 is a strong indicator of exhaustion. Clusters 10 and 13 are characterized by an increased expression of the activation markers CD69 and CD38 and markers for immunosenescence CD161 and TIGIT, with cells in cluster 10 also being positive for the exhaustion marker NKG2A. This demonstrates that there are very distinct changes within the NK cell compartment during the course of an acute SARS-CoV-2 infection. Interestingly, while we could separate REC from HC samples in the PCA analysis, the NK cell subset distribution was very similar between REC and HC (**Figure 3A**). We found only minor differences in very small clusters which was consistent with our analysis in **Figure 2**.

## Discussion

By now there are several studies investigating the phenotype and the function of NK cells during COVID-19. The strength of our study is the investigation of the NK cell phenotype during the course of an acute SARS-CoV-2 infection in hospitalized patients. This phase is dominated by innate immune responses and the adaptive response is only developing as evident by the increase of antibody levels in the samples A-C. Analysis of multiomics data from several cohorts revealed strong effects of increased TGF, TNF and IFN signaling in response to SARS-CoV-2 infection on NK cell function and phenotype (Kramer et al., 2021; Witkowski et al., 2021). Importantly, similar to our findings, severe COVID-19 was also associated with strongly increased protein expression of the activation markers CD38, CD69, and HLA-DR in early disease (Bjorkstrom and Ponzetta, 2021; Kramer et al., 2021).

Using PCA, we were able to distinguish samples from acutely infected, recovered and healthy controls. The REC samples were located between the extreme groups, indicating a gradual regeneration of the phenotype after recovery from acute disease. In line with previous reports, separation of the groups by PC1 was based in particular on the expression of the activation markers CD69, CD38 and HLA-DR and elevated signs of exhaustion with increased PD-1, TIGIT, and CTLA-4 and low CD16 and CD161. Therefore, NK cells scoring high for PC1 can be considered as highly activated and exhausted. Modulation of exhaustion markers has been described in the context of COVID-19 (Bjorkstrom and Ponzetta, 2021), but also other viral infections (Roe, 2022). CD161 marks NK cells highly responsive to cytokines and is down-modulated upon CMV infection in healthy and HIV-infected subjects (Kurioka et al., 2018). A recent report describes strong upregulation of the CD161 ligand LLT1 during SARS-CoV-2 infection, which results in impairment of NK cell effector functions (Lenart et al., 2023). Although PC1 scores of REC samples are comparable to those of HC, the majority of REC (but not HC) samples score high for PC3, which is characterized by high loadings of the senescence markers CD57 and KLRG1. This indicates a long-lasting change of NK cell properties, which has not completely normalized even after 2 months similar to other reports (Herrera et al., 2022).

Separation of the three visits by PCA failed, which can be explained by the rather small differences between samples taken at the different time points (which were at days 1, 4 and 7 of hospitalization). While we found a trend toward lower levels of CD69 and CD38, the expression of most individual markers was stable over the time of hospitalization. Also within the group of hospitalized patients there were differences in severity of COVID-19 with some patients requiring ICU care, ventilation, and two patients died from the infection. However, these subgroups were too small to perform any additional meaningful analysis.

In a conventional analysis, we only found small yet significant changes in NKG2A expression, which is in contrast to an early report (Zheng et al., 2020b) describing upregulation of NKG2A on exhausted NK cells. However, later studies only showed small effects or even found no change in NKG2A expression levels consistent with our results (Bozzano et al., 2021; Varchetta et al., 2021). In fact, we found comparably low expression levels of NKG2A at visit A, which only slightly increased over the course of the disease. These small effects can be explained by the gradual enrichment of NKG2A expressing CD56^bright^ NK cells of Phenograph cluster 6 from visit A to C and the general overrepresentation of NKG2A^hi^ clusters 10 and 12 in COV samples compared to HC. Recently, the HLA-E stabilizing peptide SARS-CoV-2 Nsp13 was shown to abrogate HLA-E/NKG2A interaction and NK cell inhibition, resulting in effective control of SARS-CoV-2 by missing self-recognition (Hammer et al., 2022). One can therefore speculate that the presence of this peptide might facilitate the activation and expansion of NKG2A^+^ cells in our samples. However, in chronic viral infections and cancer, NKG2A has also been associated with a functionally exhausted phenotype of NK cells (Bi and Tian, 2017; Sun and Sun, 2019). Phenograph cluster 2 is characterized by strong upregulation of NKG2C and CD57. This population was described to expand in CMV infection and is supposed to be involved in control of CMV infection, but also exhibits adaptive features (Kared et al., 2018). Interestingly, the deletion of the NKG2C gene increases the risk for severe course of SARS-CoV-2 infection (Vietzen et al., 2021). The presence of CD57^+^ NKG2C^+^ NK cells was associated with SARS-CoV-2 specific responses in recovered subjects (Herrera et al., 2022), but also with prolonged immune response in long-COVID (Galan et al., 2022). The strong upregulation of this subset in our COV samples may therefore indicate an effective immune response to SARS-CoV-2, but possibly also the emergence of a SARS-CoV-2-specific NK cell memory.

Our study also has certain limitations. While we use two different control groups, our study was performed in a single center and was only designed as an observational study. Therefore, we cannot provide a potential mechanism for the changes we observed, and while many of our findings are consistent with existing studies, they will need to be replicated in future studies of respiratory infections.

Taken together, we find evidence that NK cells are involved in the early innate immune response against SARS-CoV-2. Within the NK cell compartment we find an expansion of highly activated and adaptive NK cell subsets in hospitalized COVID-19 patients. We also find an increase in NK cell subsets with the expression of exhaustion and senescent markers and changes within the NK cell compartment that may still persist for some time after recovery from SARS-CoV-2 infection.

## Data availability statement

The raw data supporting the conclusions of this article will be made available by the authors, without undue reservation.

## Ethics statement

The studies involving humans were approved by #301-2008, CAPNETZ, Hannover Medical School and #178, IfADo. The studies were conducted in accordance with the local legislation and institutional requirements. The participants provided their written informed consent to participate in this study.

## Author contributions

MC: Conceptualization, Data curation, Formal Analysis, Investigation, Methodology, Writing – original draft. NP: Investigation, Writing – review & editing. DU: Investigation, Methodology, Writing – review & editing. PB: Formal Analysis, Writing – review & editing. BS: Conceptualization, Resources, Writing – review & editing. DD: Resources, Writing – review & editing. FR: Resources, Writing – review & editing. CW: Conceptualization, Supervision, Writing – original draft.
