# Supplementary material for: Early expansion of activated adaptive but also exhausted NK cells during acute severe SARS-CoV-2 infection
Source: Front Cell Infect Microbiol. 2023 Aug 30;13:1266790. doi: 10.3389/fcimb.2023.1266790 (PMC10499356; doi:10.3389/fcimb.2023.1266790)
Supplement: Supplementary file 1 [file DataSheet_1.pdf]

Early expansion of activated adaptive but also exhausted NK cells during acute severe SARS-CoV-2 infection

Maren Claus[1], Naomi Pieris[1], Doris Urlaub[1], Peter Bröde[1], Bernhard Schaaf[2, 3], Deniz Durak[4], Frank Renken[4], Carsten Watzl[1]*

[1]Department for Immunology, Leibniz Research Centre for Working Environment and Human Factors (IfADo) at TU Dortmund, Dortmund, Germany.
[2] Department of Respiratory Medicine and Infectious Diseases, Klinikum Dortmund, Dortmund, Germany.
[3] Faculty of Health, University Witten/Herdecke, Herdecke, Germany.
[4]Dortmund Health Department, Dortmund, Germany.

Supplementary Material

*Address correspondence to:

Carsten Watzl
watzl@ifado.de

Leibniz Research Centre for Working Environment and Human Factors (IfADo) at TU Dortmund
Ardeystrasse 67
44139 Dortmund, Germany

Supplementary Tables

Suppl. Table 1: Antigens, antibody clones and coupled fluorochromes, reagents, distributors and Ab dilution used to stain 0.5 × 10^6 PBMC are listed.

Target	clone	Fluorochrome	Peak Emission Channel	distributor	dilution 1/x
CD38	HB7	BUV395	UV2	BD Biosciences	100
CD159c (NKG2C)	134591	BUV496	UV7	BD Biosciences	50
CD3	UCHT1	BUV563	UV9	BD Biosciences	200
CD16	3G8	BUV615	UV10	BD Biosciences	400
CD161	HP-3G10	BUV661	UV11	BD Biosciences	100
CD32	FLI8.26	BUV737	UV14	BD Biosciences	50
CD56	B159	BUV805	UV16	BD Biosciences	500
CD137 (4-1BB)	4B4-1	BV421	V1	Biolegend	50
CD4	RPA-T4	V450	V3	BD Biosciences	200
CD64	10.1	BV480	V5	BD Biosciences	200
KLRG1	2F1	BV510	V7	Biolegend	50
CD45	HI30	BV570	V8	Biolegend	400
HLA-DR	L243	BV605	V10	Biolegend	50
CD19	HIB19	BV650	V11	Biolegend	200
NKp44	p44-8	BV711	V13	BD Biosciences	200
CD69	FN50	BV750	V14	Biolegend	400
TIGIT	741182	BV786	V15	BD Biosciences	100
CD57	HNK-1	FITC	B2	Biolegend	400
CD8a	RPA-T8	AF532	B3	ThermoFisher Scientific	200
CD14	MφP9	BB700	B9	BD Biosciences	1000
CD27	M-T271	PerCP-Cy5.5	B9	BD Biosciences	50
CD159a (NKG2A)	Z199	PE	YG1	Beckman Coulter	100
CD152 (CTLA-4)	BNI3	PE-Dazzle	YG3	Biolegend	100
CD253 (TRAIL)	N2B2	PE-Cy7	YG9	Biolegend	50
CD279 (PD-1)	EH12.1	AF647	R2	BD Biosciences	100
CD18	TS1/18	AF700	R4	Biolegend	800
live / dead	n/a	Zombie NIR	R6	Biolegend	700
CD66b	QA17A51	APC-Fire 750	R7	Biolegend	400
True-Stain Monocyte Blocker™				Biolegend	
Brilliant Stain Buffer				BD Biosciences	
Human serum				Pan-Biotech	

Suppl. Table 2: Loadings of 3 Principal Components explaining 51.5 % of variance. NK cells from COV, REC and HC were analyzed by flow cytometry and frequency of NK cells and expression levels (as geoMean) of surface markers related to activation, exhaustion and senescence were subjected to PCA.

Variable	PC1 (25.2 %)	PC2 (16.3 %)	PC3 (10.0 %)
% NK	0.022	-0.634	0.448
CD38	0.628	0.038	0.179
CD16	-0.682	-0.003	0.311
CD161	-0.267	0.637	0.310
41BB	0.397	0.505	0.486
KLRG1	-0.622	-0.052	0.286
HLA-DR	0.577	-0.157	-0.192
CD69	0.589	0.102	0.400
TIGIT	0.287	-0.151	-0.257
CD57	0.076	-0.624	0.475
CD27	0.426	0.626	-0.327
NKG2A	0.028	0.317	-0.176
CTLA-4	0.899	-0.125	0.184
TRAIL	0.261	0.671	0.419
PD-1	0.550	-0.326	-0.130
CD8	-0.650	0.173	-0.018

Supplementary Figures

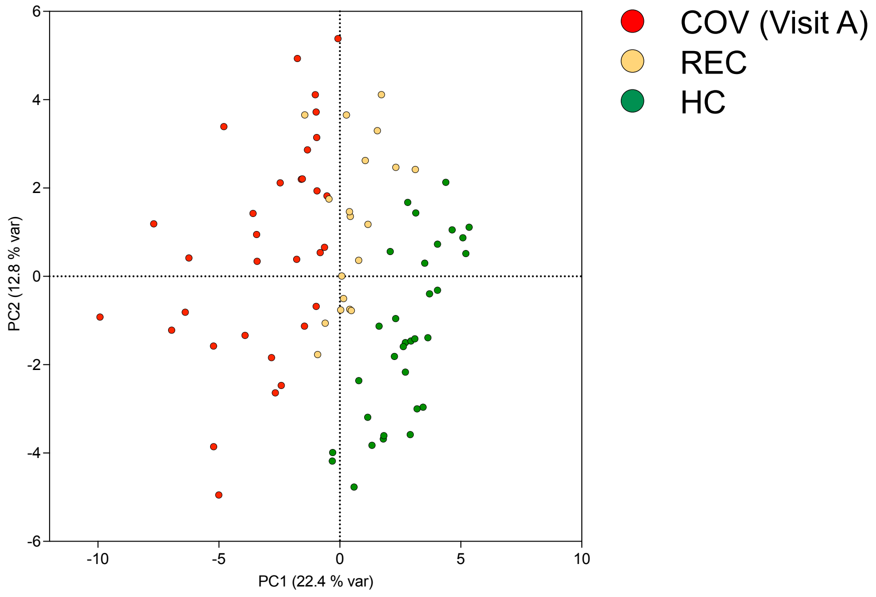

Suppl. Figure 1
Principal component analysis of selected markers expressed on lymphocytes. Scores of individual samples from COV (visit A), REC and HC for PCs 1 and 2, together explaining 35 % of variance.

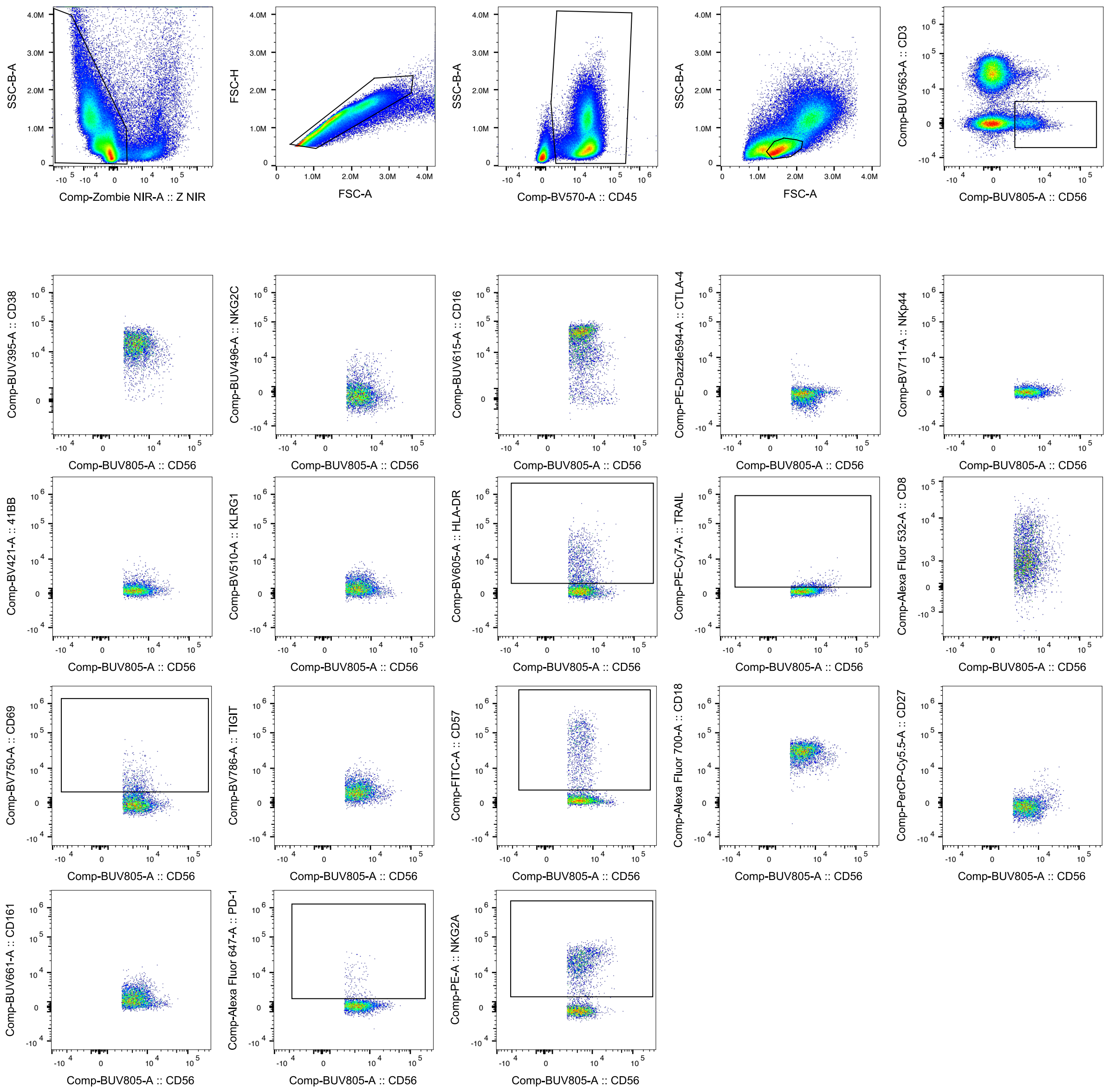

Suppl. Figure 2

Gating strategy. Lymphocytes are gated from live (Zombie NIR negative) single cells by CD45 and SSc characteristics. NK cells are CD56+ CD3- lymphocytes. Expression levels of surface markers on NK cells are determined as geometric Mean and for selected markers, frequency of positive NK cells was determined.

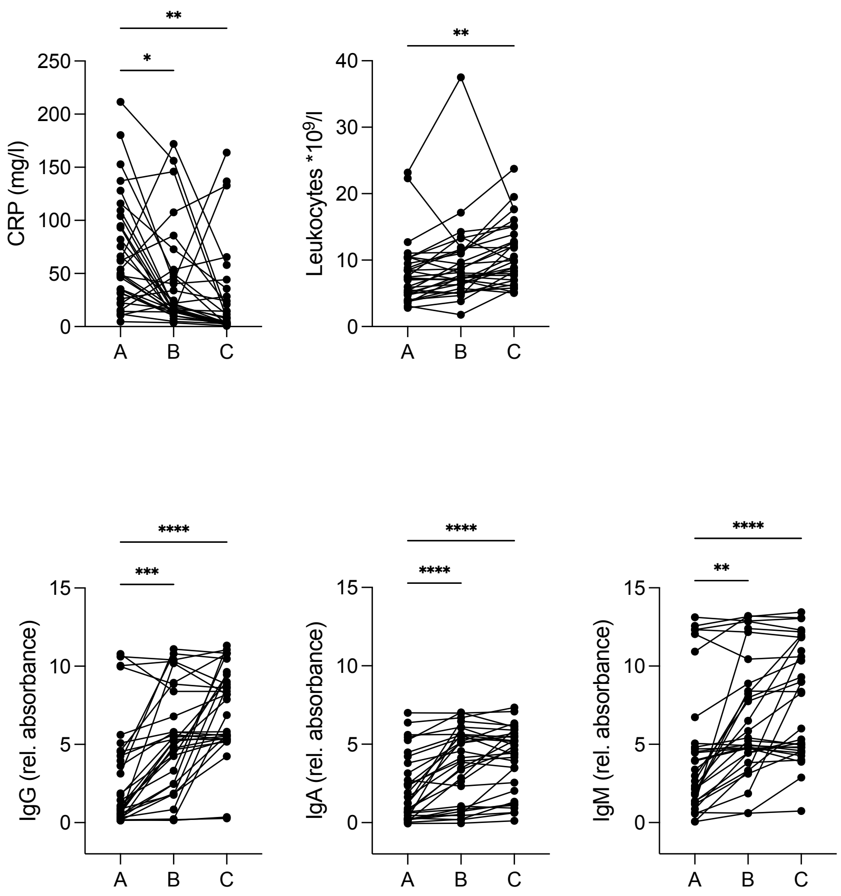

Suppl. Figure 3
Clinical parameters of the COV group. Pairwise comparisons of serum CRP concentration and leukocyte count at visits A-C were performed using a mixed-effects analysis with Tukey's post test. Pairwise comparisons of antibody concentrations at visits A-C were performed using the Friedman test with Dunn‘s post test. (*:p<.05, **:p<.01, ***:p<.001, ****:p<.0001).

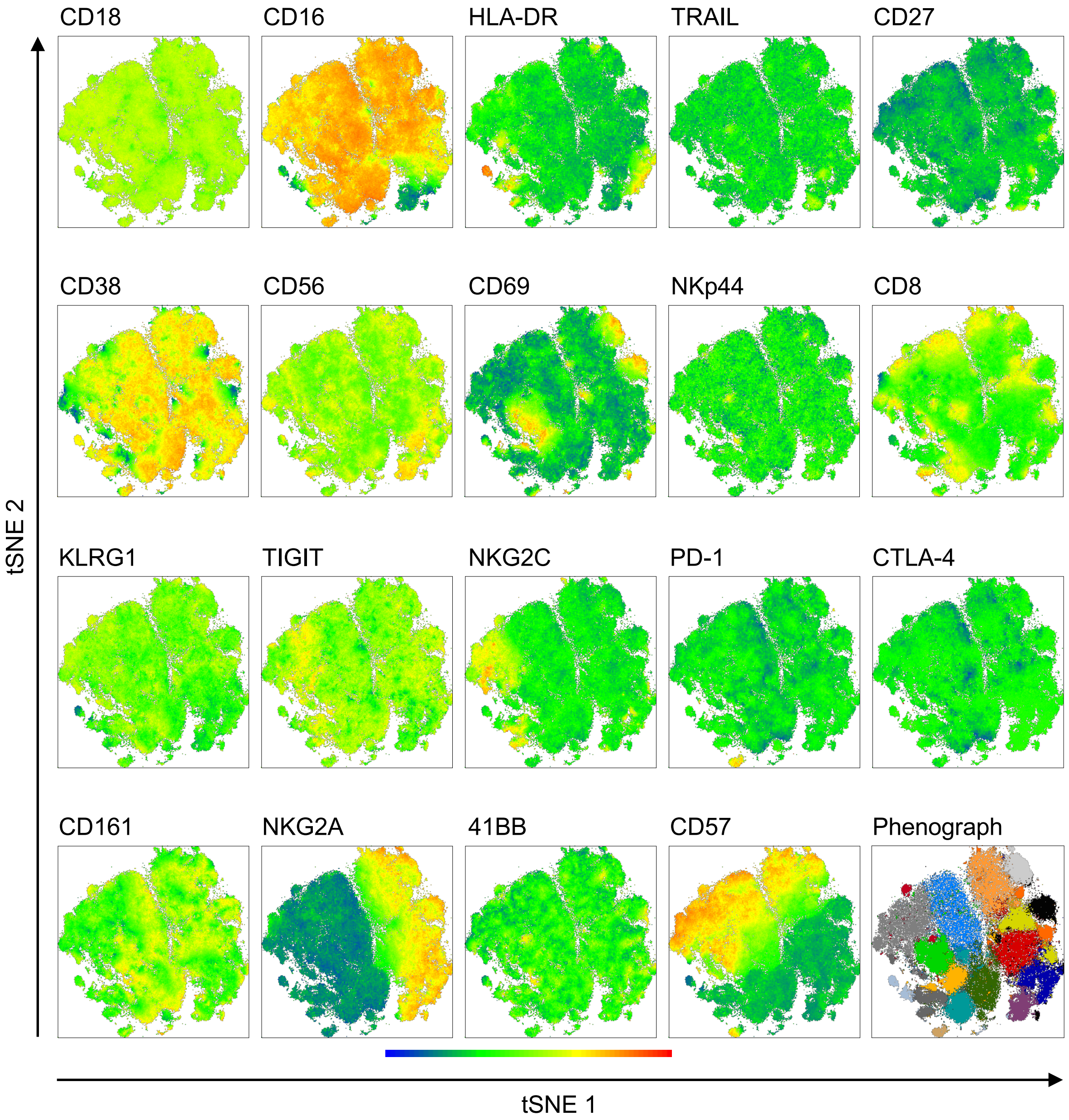

Suppl. Figure 4
Unsupervised high-dimensional analysis of NK cells. All samples were gated on NK cells and then concatenated. The concatenated dataset was subjected to dimensionality reduction by tSNE and cluster analysis using Phenograph.

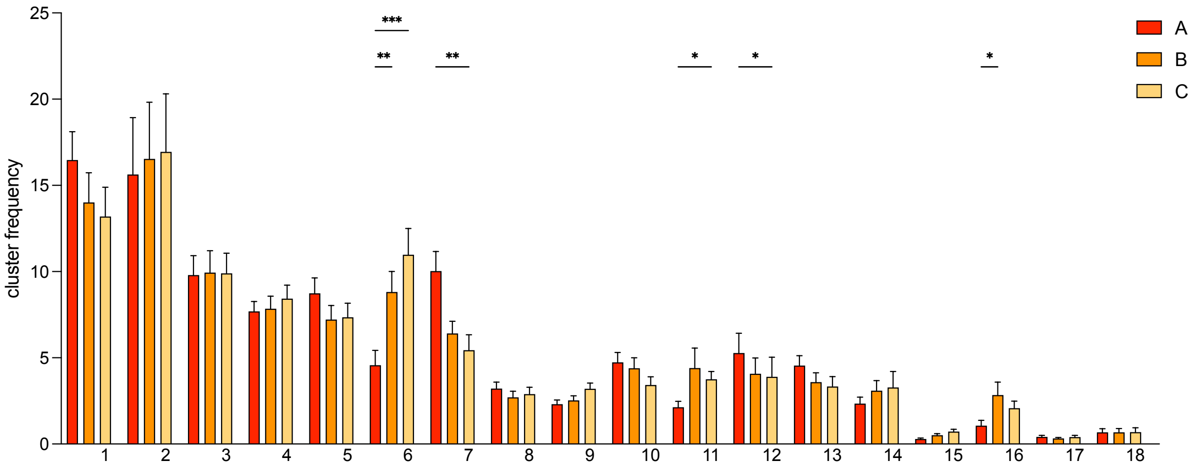

Suppl. Figure 5

Frequency distribution of Phenograph clusters in COV visits A-C. Pairwise comparisons between visits were performed using the Friedman test with Dunn‘s post test. (*:p<.05, **:p<.01, ***:p<.001).

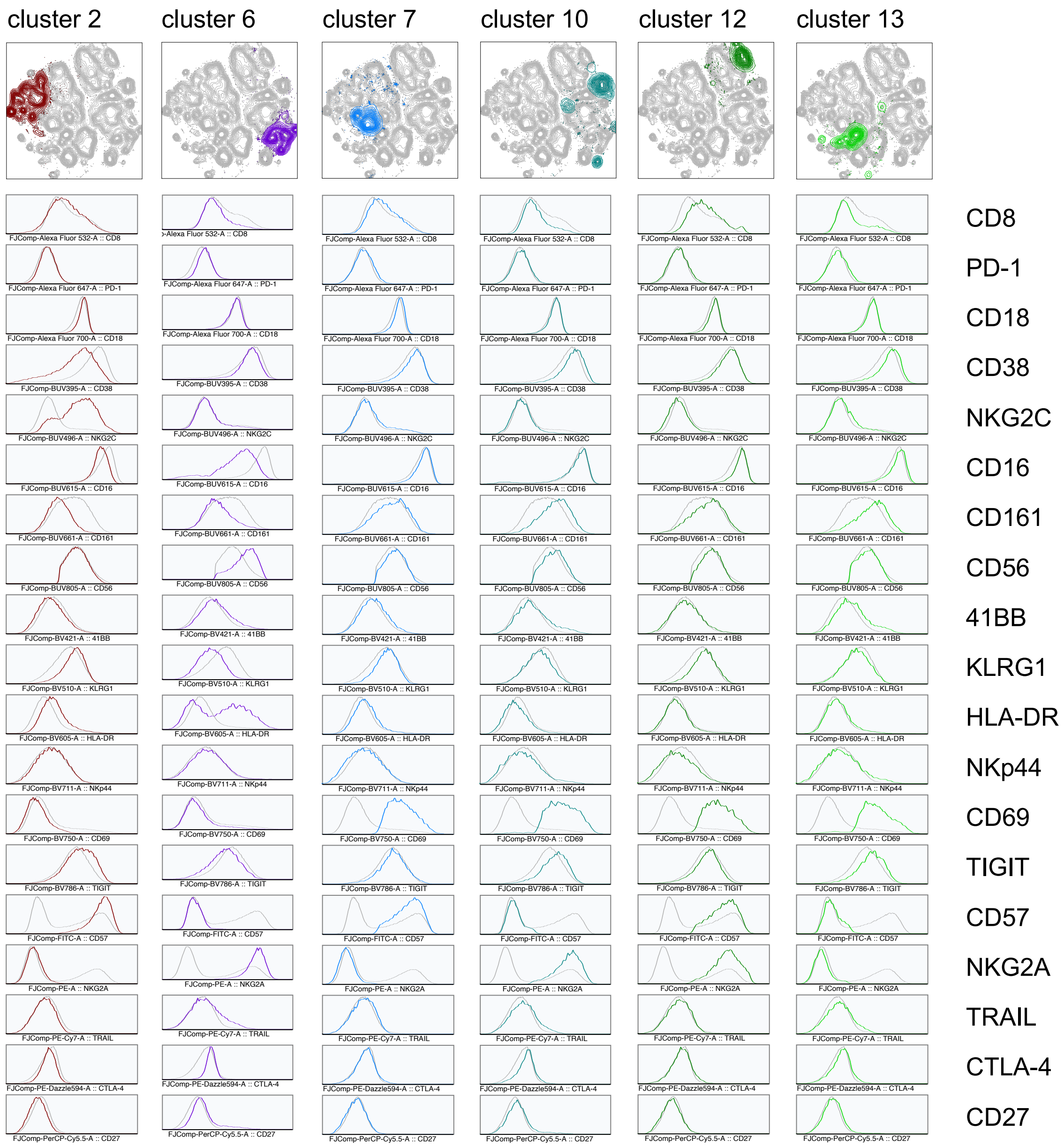

Suppl. Figure 6
Unsupervised high-dimensional analysis of NK cells. All samples were gated on NK cells and then concatenated. The concatenated dataset was subjected to dimensionality reduction by tSNE and cluster analysis using Phenograph, and Phenograph clusters overrepresented in COV visit A are shown as overlay with HC.
